# Supplementary material for: The phosphorylation of Pak1 by Erk1/2 to drive cell migration requires Arl4D acting as a scaffolding protein
Source: J Cell Sci. 2025 May 22;138(10):jcs263812. doi: 10.1242/jcs.263812 (PMC12148019; doi:10.1242/jcs.263812)
Supplement: Supplementary information [file joces-138-263812-s1.pdf]

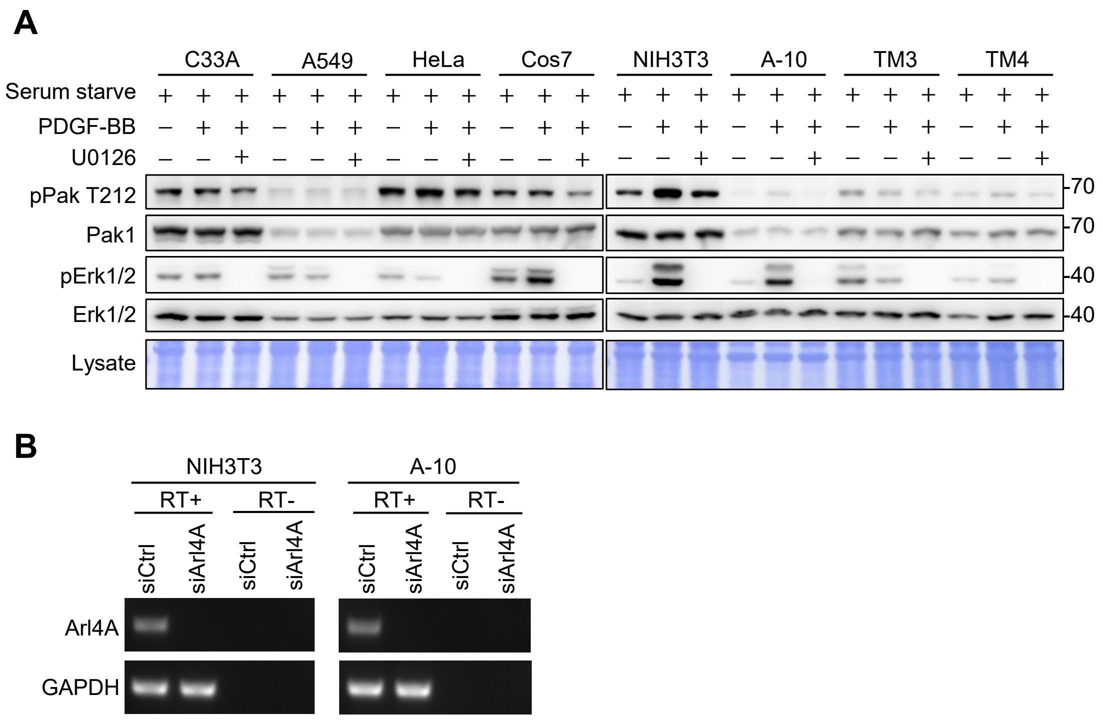

Fig. S1. Screening of PDGF sensitive cell lines and confirmation of knockdown efficiency. (A) Indicated cell lines were serum staved and then treated with PDGF-BB at 20 ng/mL for 10 min or either co-treated with U0126 (10 uM). The indicated proteins were blotted with specific antibodies. Total cell lysates were stained with Coomassie blue. (B) NIH3T3 and A-10 cell lines knocked down with siCtrl and mouse siArl4A were serum staved and then treated with PDGF-BB (20 ng/mL) for 10 min. Total RNA was extracted from these cells and then subjected to RT-PCR. Using Arl4A-specific primers, the PCR products were analyzed on a 1.5% agarose EtBr gel for UV detection representing the mRNA levels of Arl4A.

Journal of Cell Science • Supplementary information

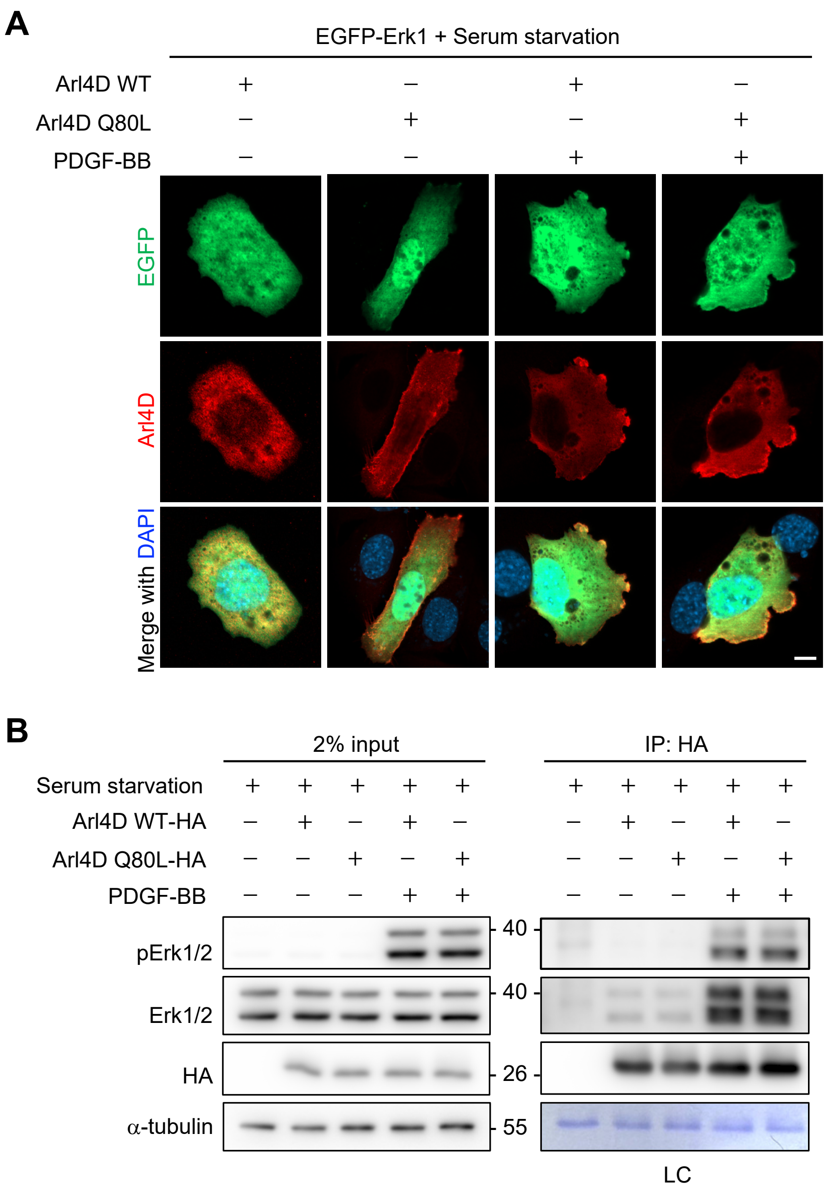

Fig. S2. Arl4D interacts with activated Erk1/2 under PDGF signaling. (A) NIH3T3 cells transfected with indicated proteins were serum starved, treated with PDGF-BB at 20 ng/mL for 0 or 10 mins and stained with anti-Arl4D (red) antibodies and DAPI (blue; stains the nuclei). Scale bar, 10 mm. (B) NIH3T3 cells transfected with empty vector pSG5, Arl4D WT-HA or Arl4D Q80L-HA were serum starved, treated with PDGF-BB (20 ng/mL) for 10 min and was subjected to DSP-crosslinker-treated Co-IP.

Journal of Cell Science • Supplementary information

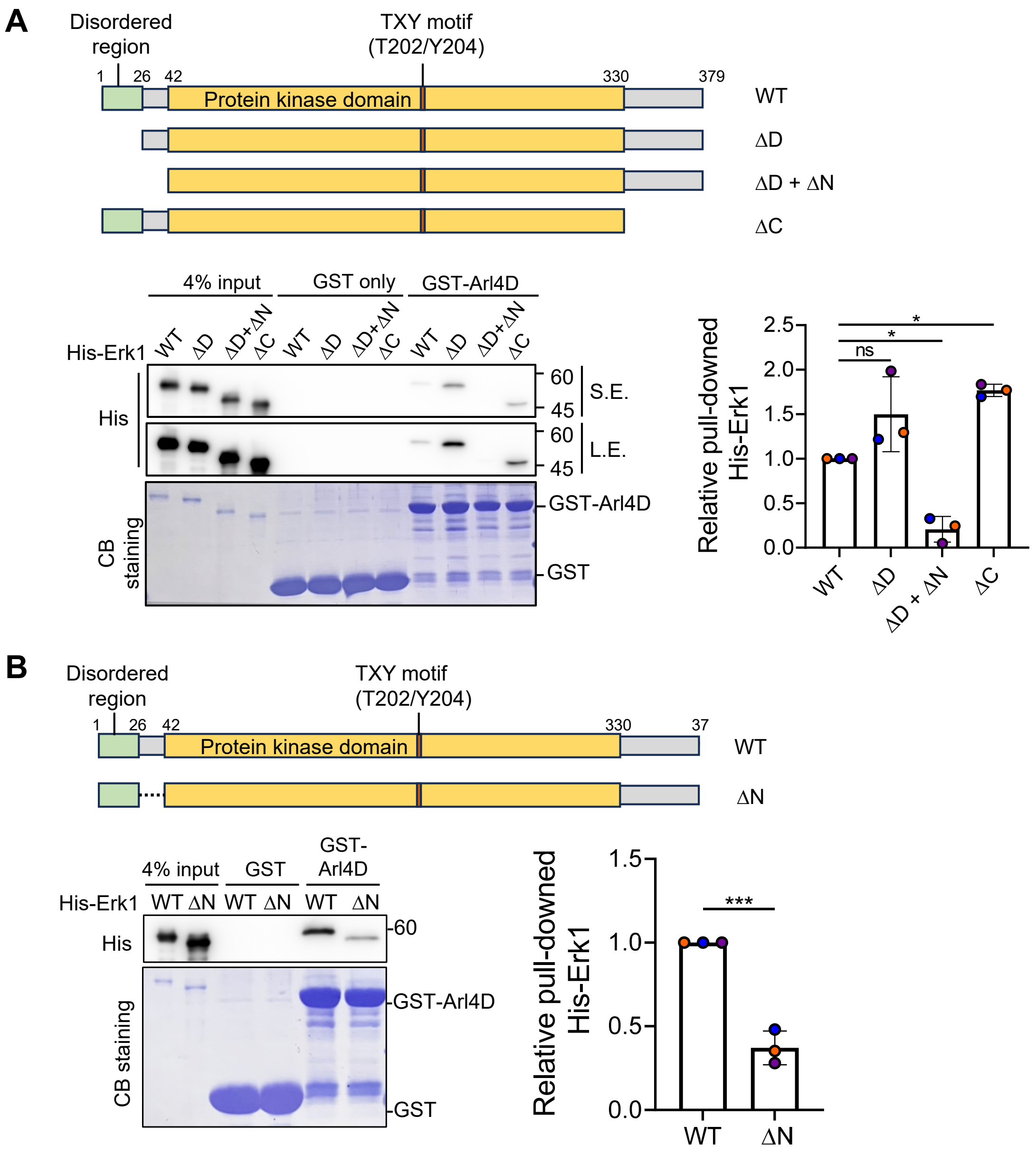

Fig. S3. Identification of Arl4D-binding-defective Erk1/2. (A and B) Cartoon representations show the Erl1 truncations (ΔD: deletion of the disorder region; ΔN: deletion of the space between the disorder region and the kinase domain; ΔC: deletion of the region downstream of the kinase domain). In vitro binding of His-tagged Erk1 proteins with GST or GST-Arl4D. His-Erk1 proteins pulled down from GST fusion proteins were analyzed by Western blotting. Equal amounts of GST proteins were detected by staining with CB (Coomassie blue). The His signals of the pulled-down His-Erk1 proteins were quantified in the dot plots; the error bars indicate the mean ± (n=3). *P < 0.05; ***P < 0.001 (one-way ANOVA with Tukey's post-hoc multiple comparison test in (A), two-tailed unpaired Student's t-test in (B)).

Journal of Cell Science • Supplementary information

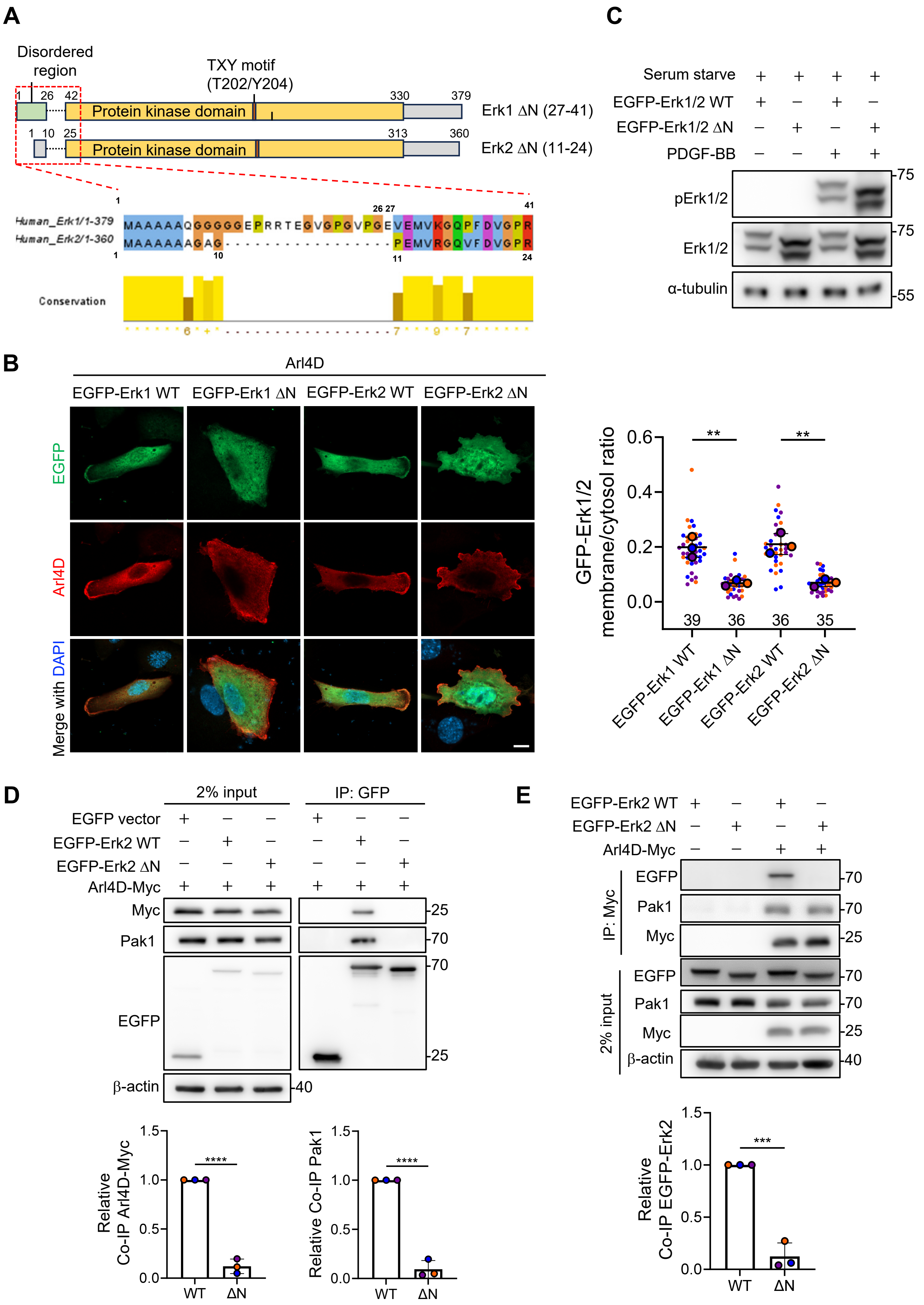

Journal of Cell Science • Supplementary information

Fig. S4. Erk1 and Erk2 share the same N-terminal region to interact with Arl4D. (A) Jalview software shows the alignment and conservation between Erk1 and Erk2. (B) NIH3T3 cells transfected with the indicated proteins were stained with anti-Arl4D antibodies (red) and DAPI (blue; stains the nuclei). Scale bar, 10 mm. The ratio between plasma membrane and cytosol of GFP-Erk1/2 in each group was quantified as described in Materials and Methods, and the results were shown in the dot plots with error bars indicating the mean ± SD (n=3, the cells analyzed in each biological replicate were marked in the same color, and the total number of cells is indicated in the graph). **P<0.01 (one-way ANOVA with Tukey's post hoc multiple comparison test). (C) NIH3T3 cells transfected with EGFP-Erk1/2 WT or EGFP-Erk1/2 ΔN were serum starved and treated with PDGF-BB (20 ng/mL) for 10 min. Immediately harvested cell lysates were subjected to Western blotting for the indicated proteins. (D) NIH/3T3 cells transfected with pEGFP-C3, Arl4D-WT-Myc, EGFP-Erk2 WT, or EGFP-Erk2 ΔN were subjected to Co-IP. The Co-IP signals of endogenous Pak1 and Arl4D-Myc were normalized with GFP-Erk2 proteins after subtracting the background signal of the pEGFP-C3 group. (E) NIH/3T3 cells transfected with empty vector pSG5, Arl4D-Myc, EGFP-Erk2 WT or EGFP-Erk2 ΔN were subjected to Co-IP. The Co-IP signals of EGFP-Erk2 were normalized with Arl4D-WT-Myc proteins after subtracting the background signal of the empty vector control group. (D and E) The quantified results are shown in the dot plots, with the error bars indicating the mean ± SD (n=3). ***P<0.001; ****P < 0.0001 (two-tailed unpaired Student's t-test).

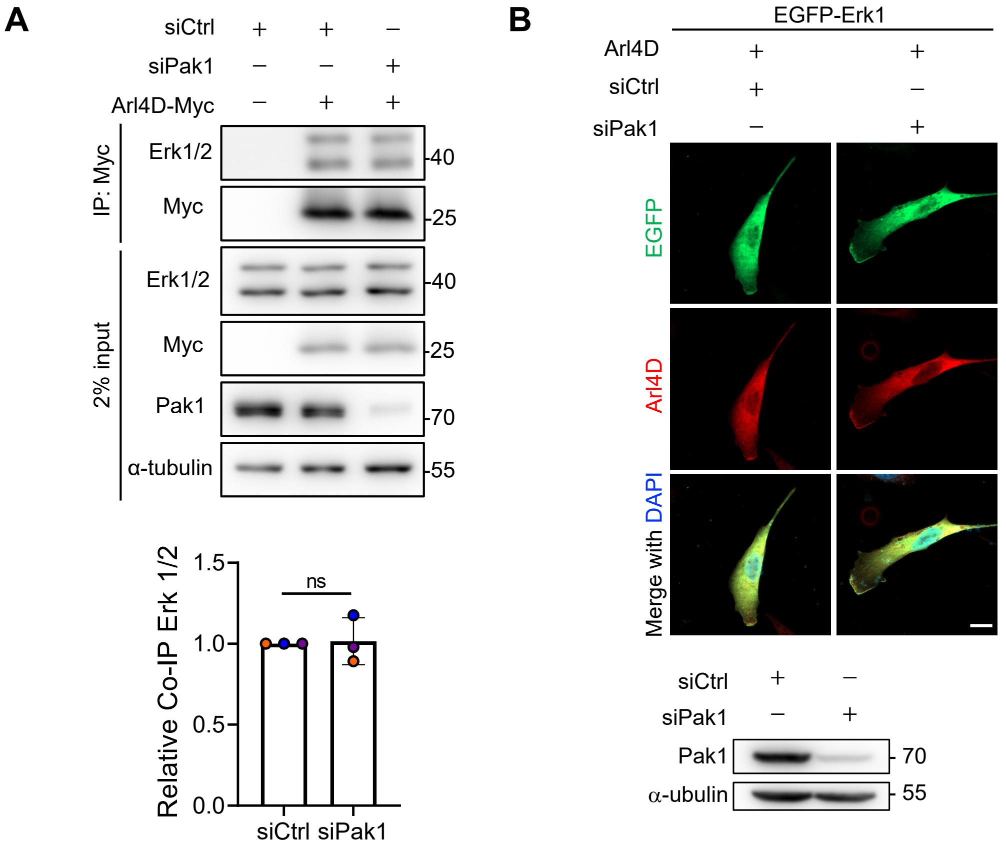

Fig. S5. Arl4D interacting with Erk1/2 is independent of Pak1. (A) NIH3T3 cells knocked down with siCtrl or mouse siPak1 RNA and transfected with the empty vector pSG5 or Arl4D-Myc were subjected to Co-IP. The Co-IP signals of the endogenous Erk1/2 proteins were normalized with the Arl4D-Myc proteins after subtracting the background signal of the empty vector control group. The quantified results are shown in the dot plots with error bars indicating the mean ± SD (n=3, two-tailed unpaired Student's t-test). (B) NIH/3T3 cells knocked down with siCtrl or mouse siPak1 and transfected with the indicated proteins were stained with anti-Arl4D antibodies (red) and DAPI (blue; stains the cell nuclei). Scale bar, 10 mm. The right panel shows the expression level of indicated protein.

Journal of Cell Science • Supplementary information

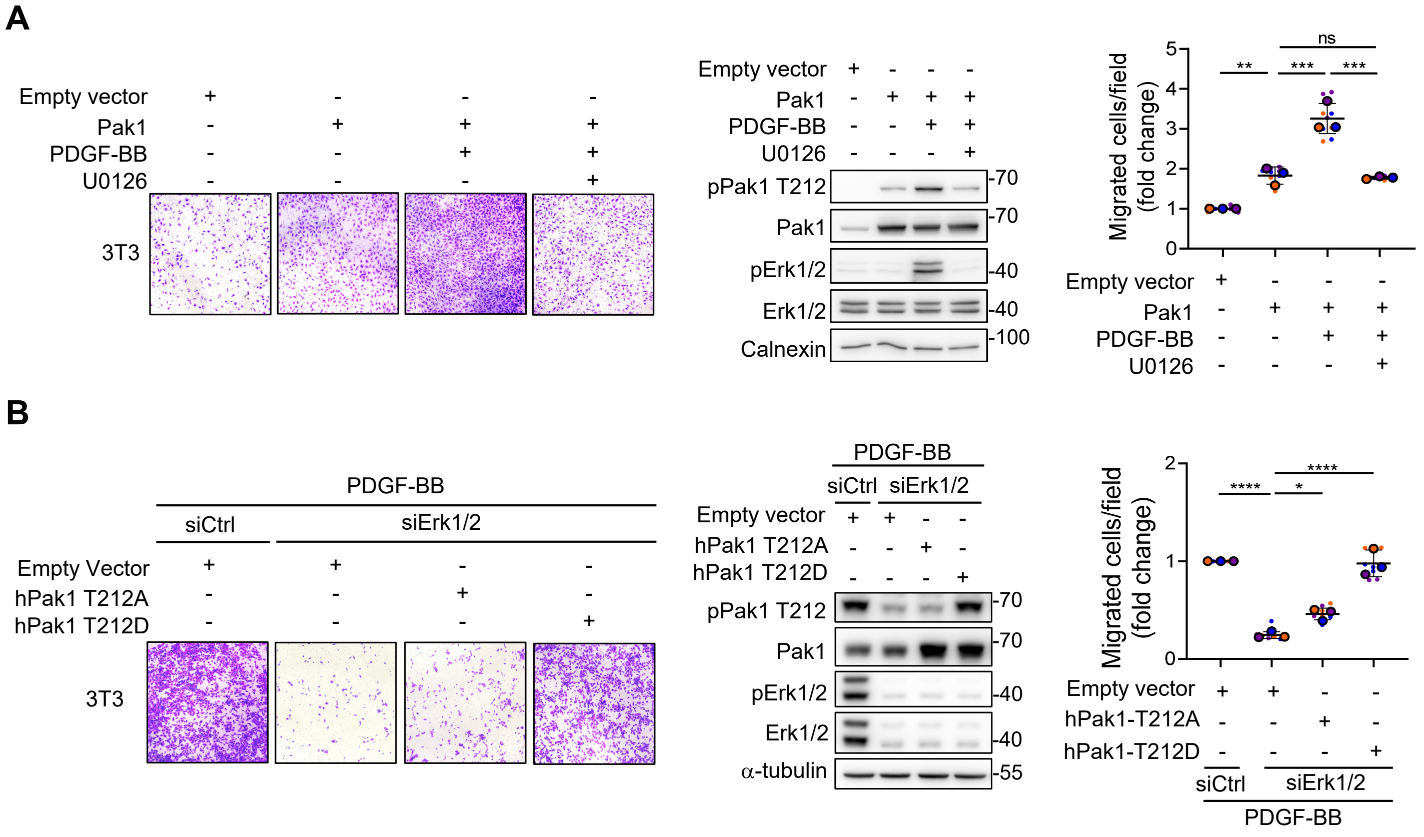

Fig. S6. Pak1 T212 phosphorylation is important downstream signal under PDGF-Erk1/2 to promote cell migration. (A) NIH3T3 cells transfected with empty vector pcDNA3.0 or Pak1 were serum starved and treated with PDGF-BB (20 ng/ml) or co-treated with U0126 (10 uM). Western blotting detected the indicated proteins. (B) NIH3T3 were knocked down with siCtrl or mouse siErk1/2 RNA and transfected with empty vector pcDNA3.0, hPak1-T212A or hPak1-T212D. The cells were kept serum-free and treated with PDGF-BB (20 ng/ml). Western blotting detected the indicated proteins. (A and B) Cells migrated under the transwells were stained with crystal violet and photographed for 3 fields per biological replicate and calculated in dot plot with mean ± SD (n=3). *P<0.05; **P<0.01; ***P<0.005; ****P<0.001 (one-way ANOVA with Tukey's post hoc multiple comparison test).

Journal of Cell Science • Supplementary information

Figure 1A

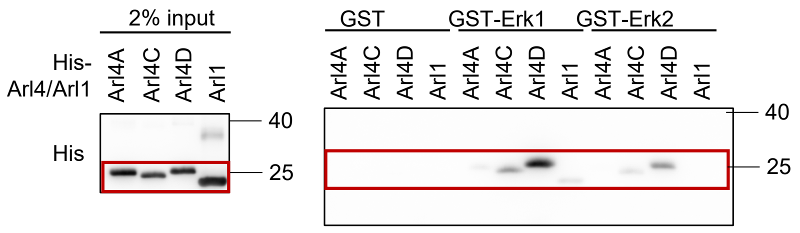

Figure 1C

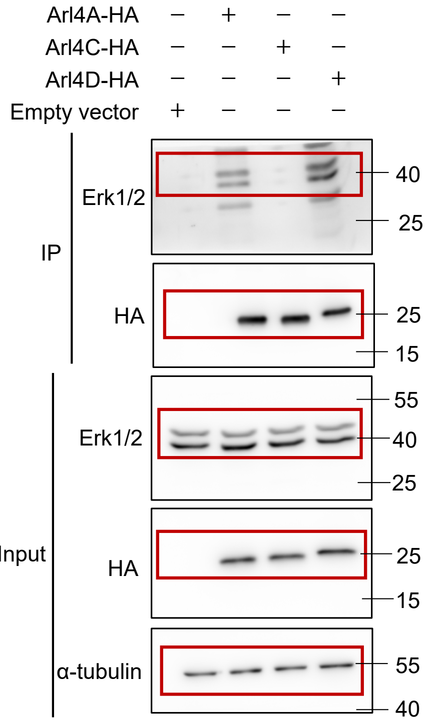

Figure 2A

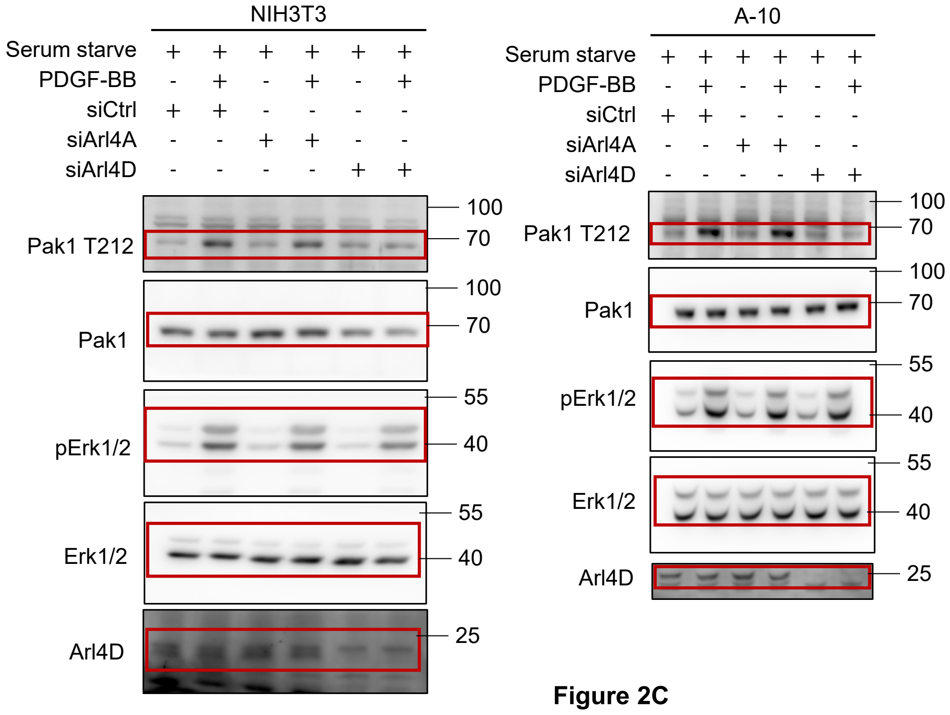

Figure 2C

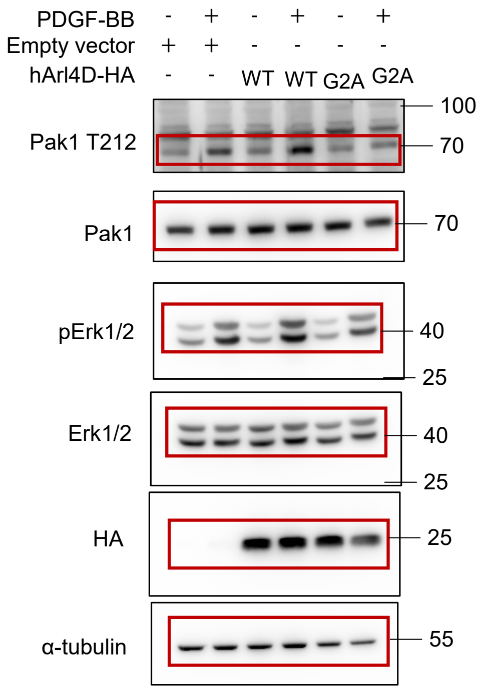

Figure 2B

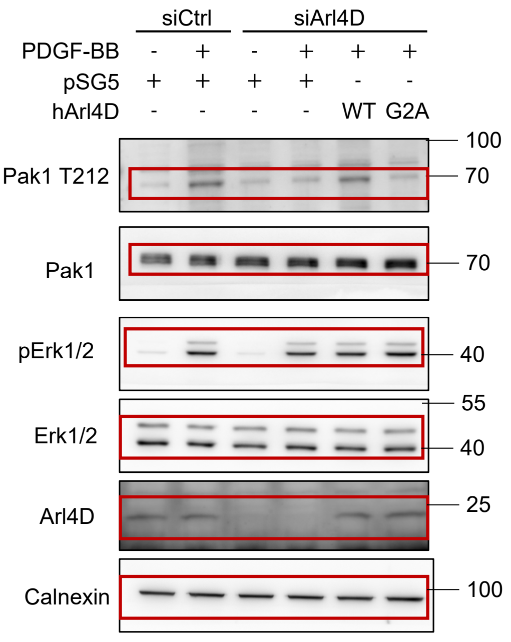

Journal of Cell Science • Supplementary information

Figure 3B

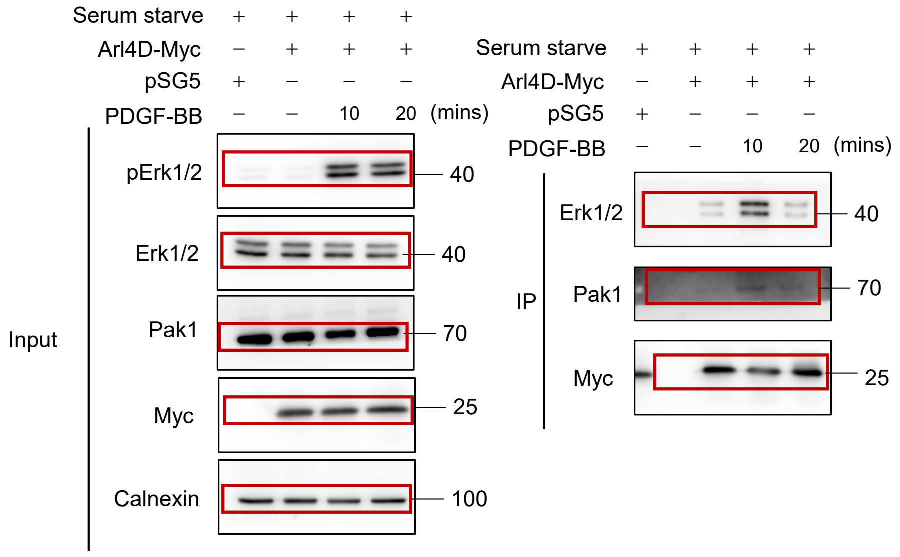

Figure 3C

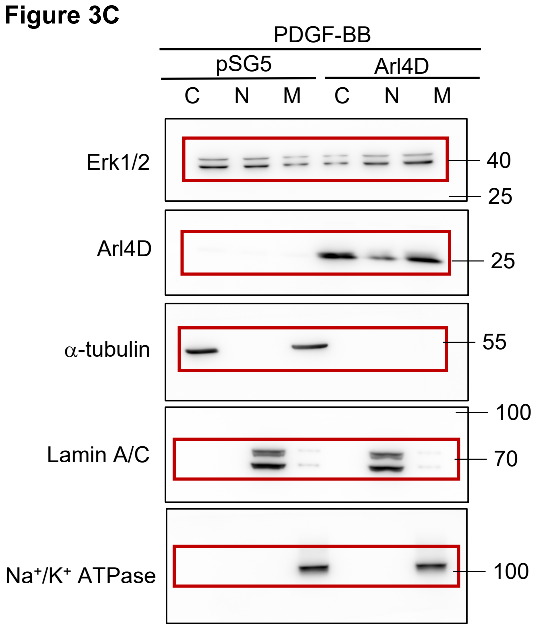

Figure 3D

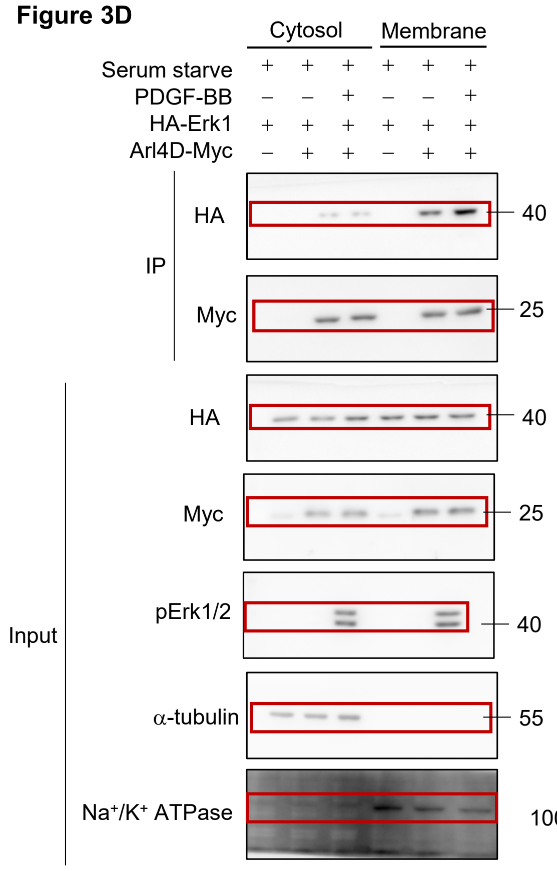

Figure 4A

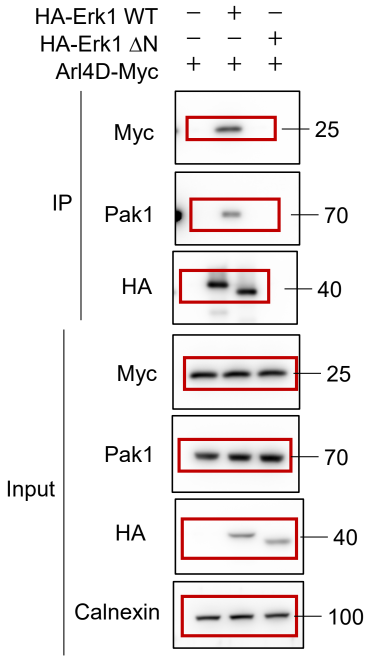

Figure 4B

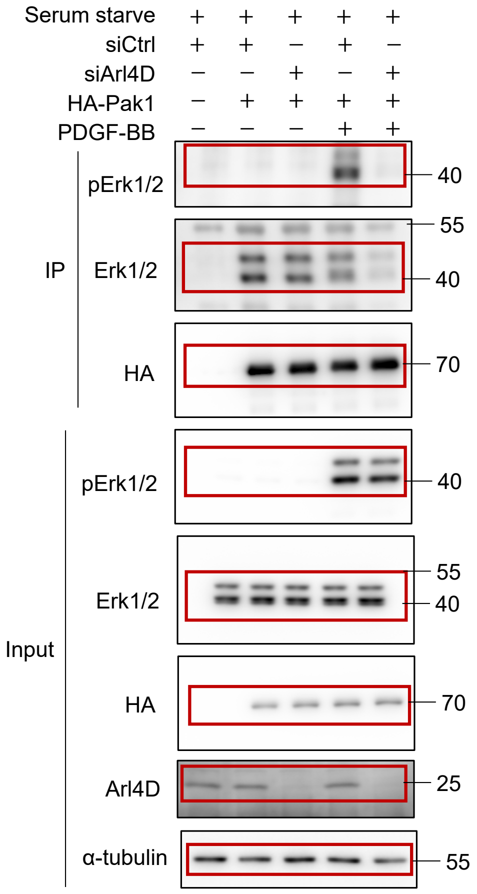

Figure 4C

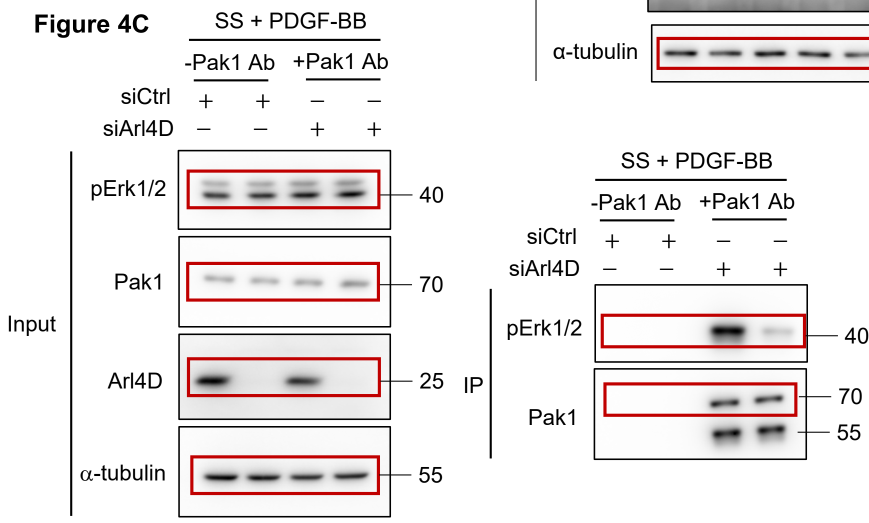

Figure 4D

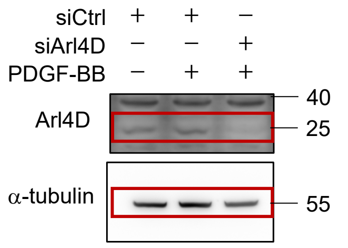

Journal of Cell Science • Supplementary information

Figure 4E

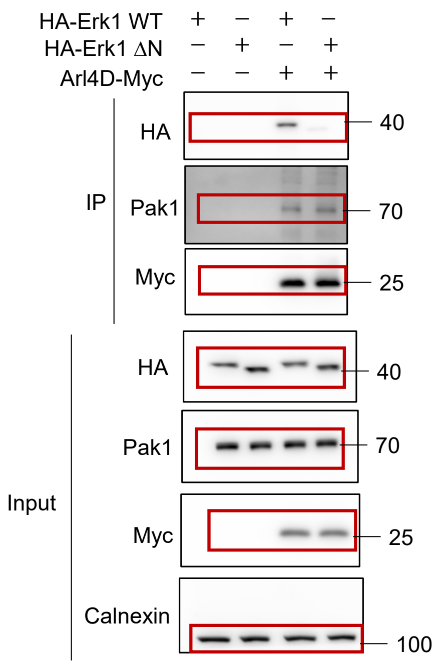

Figure 5A

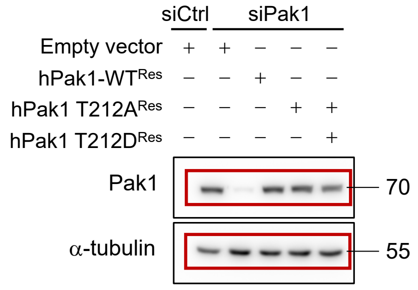

Figure 5B

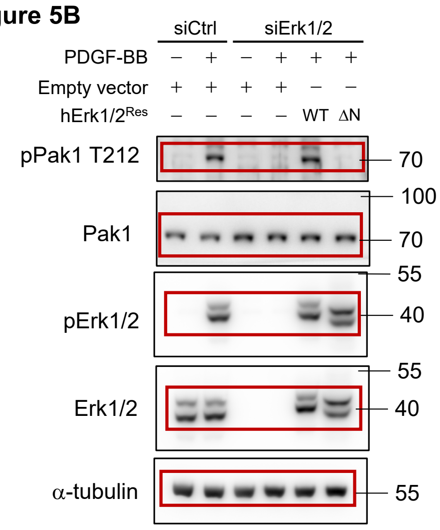

Figure 5C

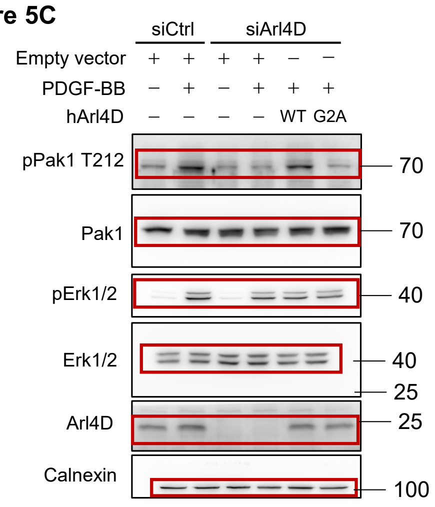

Figure S1A

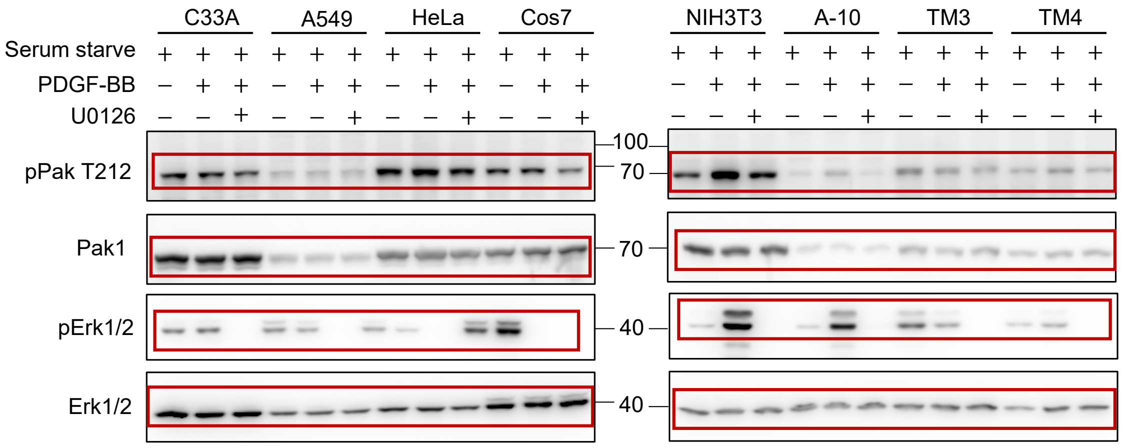

Figure S2B

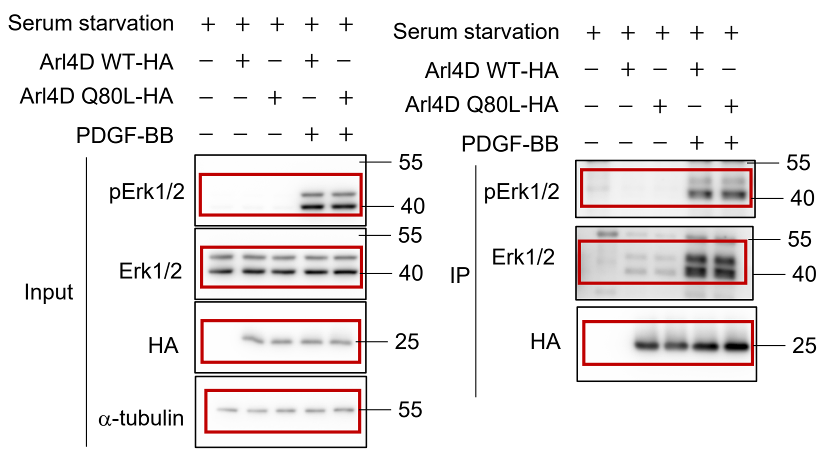

Figure S3A

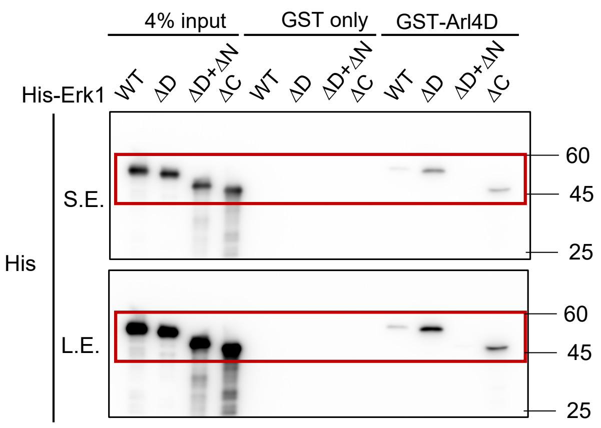

Journal of Cell Science • Supplementary information

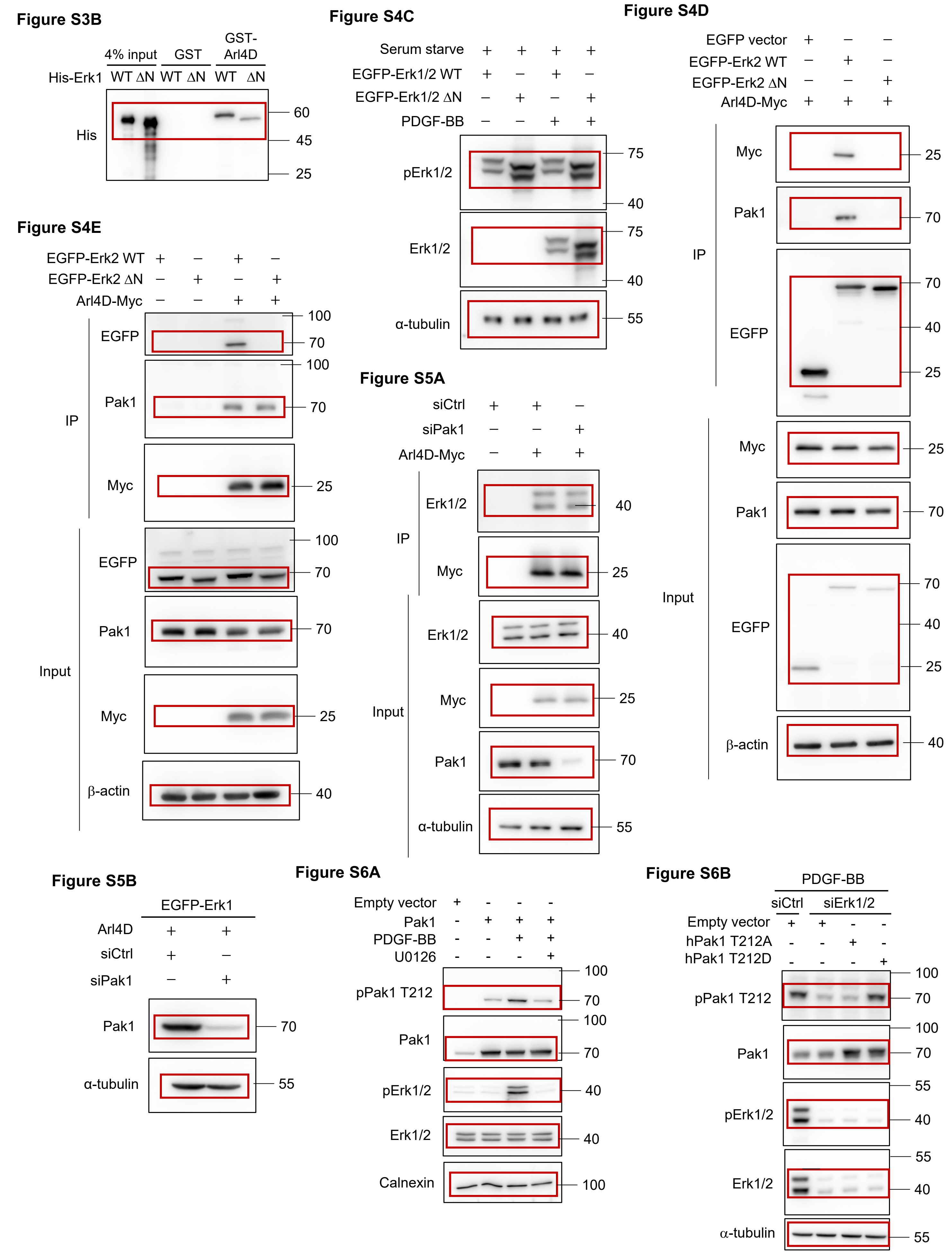

Fig. S7. Blot Transparency. Show the original image of Western Blotting.

Table S1. List of proteins specifically interacting with Arl4D QL or TN in the liposome floating experiment

Available for download at
https://journals.biologists.com/jcs/article-lookup/doi/10.1242/jcs.263812#supplementary-data
