# Supplementary material for: The phosphorylation of Pak1 by Erk1/2 to drive cell migration requires Arl4D acting as a scaffolding protein
Source: J Cell Sci. 2025 May 22;138(10):jcs263812. doi: 10.1242/jcs.263812 (PMC12148019; doi:10.1242/jcs.263812)
Supplement: Supplementary file 1 [file joces-138-263812_review_history.pdf]

Journal of Cell Science

The phosphorylation of Pak1 by Erk1/2 to drive cell migration requires Arl4D acting as a scaffolding protein

Ting-Wei Chang, Ming-Chieh Lin, Chia-Jung Yu and Fang-Jen S. Lee
DOI: 10.1242/jcs.263812

Editor: Simon J Cook

Review timeline
Original submission: 23 December 2024
Editorial decision: 27 February 2025
First revision received: 19 March 2025
Accepted: April 8 2025

Original submission

First decision letter

MS ID#: jcs.263812

MS TITLE: The phosphorylation of Pak1 by Erk1/2 for cell migration requires Arl4D acting as a scaffolding protein

AUTHORS: Ting-Wei Chang; Ming-Chieh Lin; Chia-Jung Yu; FANG-JEN S LEE

ARTICLE TYPE: Research Article

Dear Dr Lee,

We have now reached a decision on the above manuscript.

As you will see, the reviewers like your study but raise a number of substantial criticisms that prevent me from accepting the paper at this stage. They suggest, however, that a revised version might prove acceptable, if you can address their concerns including further microscopy work (rev 2) and some editorial revisions. If you think that you can deal satisfactorily with the criticisms on revision, I would be pleased to see a revised manuscript. We would then return it to the reviewers.

Reviewer 1

Advance summary and potential significance to field

It has been known for a long time that activated ERK alters cytoskeletal structures, but the mechanisms aren't clear. This ms links ERK to phosphorylation of PAK1 (at T212), via a scaffold protein ArlD4 at the plasma membrane. If true, this would add a piece to the puzzle regarding ERK actions.

The data are clear and convincing, as far as they go. However, there are some problems with the ms as written.

1) The phosphorylation of PAK1 at T212 (by CDK5) was first described in 2002, by the Chernoff and the Nicolic groups. These papers should be cited. Importantly, they found that this phosphorylation did not activate PAK1, which makes the link to cytoskeletal changes mysterious. The authors should

© 2025. Published by The Company of Biologists under the terms of the Creative Commons Attribution License (https://creativecommons.org/licenses/by/4.0/).

discuss how they think this non-activating phosphorylation leads to cytoskeletal changes in their system.

2) As far as I can tell, all their evidence for interaction comes from exogenous expression studies using tagged proteins. At least one example should be shown using endogenous proteins.

Reviewer 2

Advance summary and potential significance to field

The authors provide convincing and very interesting data to support the notion that Arl4D works as a scaffolding protein. This function enables activated Erk1/2 to target Pak1 for phosphorylation, an important determinant of cell migration. One of the most interesting features of the study is that scaffolding proteins are required for translocation of Erk from the plasma membrane to other sites in the cell. The advance in this paper is that Arl4D acts as a scaffold protein in order that activated Erk1/2 can target Pak1 at the plasma membrane. The more general idea that is interesting in this paper is that scaffolding is a requirement for substrate targeting by Erk.

Comments for the author

The paper is a very good example of concise and impactful writing. The biochemical assays are clear and cleanly conducted and the lay-out of the figures makes it easy to see what is going on with the proposed scaffolding function. The one caveat that this reviewer found with the paper is that the microscopy which is intended to show enrichment of certain proteins at the plasma membrane, and which is important for the demonstration of the idea of membrane targeting and enrichment, does not actually seem to show the expected enrichment and the data visual data do not seem to align well with the Pearson correlation coefficient. Notably, some of the plasma membrane imaging is clear (e.g., Supplementary figures S2A, S4B) while in the main figures, the enrichment is very difficult to see (Fig. 3A, 4C). If the authors were able to provide more convincing figures, this paper would be improved.

First revision

Author response to reviewers' comments

Replies to Reviewers

Reviewer #1 (Remarks to the Author):

It has been known for a long time that activated ERK alters cytoskeletal structures, but the mechanisms aren't clear. This ms links ERK to phosphorylation of PAK1 (at T212), via a scaffold protein ArlD4 at the plasma membrane. If true, this would add a piece to the puzzle regarding ERK actions.
The data are clear and convincing, as far as they go. However, there are some problems with the ms as written.

1) The phosphorylation of PAK1 at T212 (by CDK5) was first described in 2002, by the Chernoff and the Nicolic groups. These papers should be cited. Importantly, they found that this phosphorylation did not activate PAK1, which makes the link to cytoskeletal changes mysterious. The authors should discuss how they think this non-activating phosphorylation leads to cytoskeletal changes in their system.

Response: We appreciate the reviewer's comment. Two different teams discovered that CDK5 or cyclin B1/Cdc2 mediates the Pak T212 phosphorylation first in 2001 by Nicolic group (*Journal of Biological Chemistry*) and then 2002 by Chernoff group (*Current Biology*), which were important

© 2025. Published by The Company of Biologists under the terms of the Creative Commons Attribution License (https://creativecommons.org/licenses/by/4.0/).

findings to shed light on possible Pak1-driven signals during cell division. We have cited these papers and added a paragraph discussing their findings in the discussion section as follows:

“Previous study showed that Pak1-T212 phosphorylation in neurons is regulated by the p35/Cdk5 kinase, which is crucial for cytoskeleton regulation during neurite outgrowth and remodeling (Rashid, Banerjee et al. 2001). Additionally, it was found that Pak1 can be phosphorylated at T212 by cyclin B1/Cdc2 in mitotic cells (Banerjee, Worth et al. 2002, Thiel, Reeder et al. 2002). These findings highlight the significance of Pak1-T212 phosphorylation in both postmitotic neurons and mitosis, suggesting a shared signaling pathway regulating diverse cellular events. Sundberg-Smith et al. noted a reduction in CDK5 kinase activity in response to PDGF signaling (Sundberg-Smith, Doherty et al. 2005), indicating that the Pak1-Erk1/2 interaction does not affect cell migration via the CDK5-regulated microtubule remodeling pathway upon PDGF stimulation. In contrast, we have found that this stimulation promotes the phosphorylation of Pak1 at T212 through interactions among Arl4D, Erk1/2, and Pak1 to promote cell migration. Thus, we conclude that different complexes formed with phosphorylated Pak1-T212 result in different effector outcomes.”

References:

Banerjee, M., D. Worth, D. M. Prowse and M. Nikolic (2002). "Pak1 phosphorylation on t212 affects microtubules in cells undergoing mitosis." Current Biology **12**(14): 1233-1239.
Rashid, T., M. Banerjee and M. Nikolic (2001). "Phosphorylation of Pak1 by the p35/Cdk5 kinase affects neuronal morphology." Journal of Biological Chemistry **276**(52): 49043-49052.
Sundberg-Smith, L. J., J. T. Doherty, C. P. Mack and J. M. Taylor (2005). "Adhesion stimulates direct PAK1/ERK2 association and leads to ERK-dependent PAK1 Thr212 phosphorylation." Journal of Biological Chemistry **280**(3): 2055-2064.
Thiel, D. A., M. K. Reeder, A. Pfaff, T. R. Coleman, M. A. Sells and J. Chernoff (2002). "Cell cycle-regulated phosphorylation of p21-activated kinase 1." Current Biology **12**(14): 1227-1232.

2) As far as I can tell, all their evidence for interaction comes from exogenous expression studies using tagged proteins. At least one example should be shown using endogenous proteins.

Response: As requested, we conducted endogenous Co-IP experiments to support that Arl4D functions as a scaffold linking active Erk1/2 kinase to Pak1. Using NIH3T3 cells, which highly response to PDGF signal (Fig. S1A), our results demonstrated that the PDGF stimulated association of endogenous Pak1 and activated Erk1/2 is deterred by siRNA knockdown of Arl4D (new Fig. 4C below).

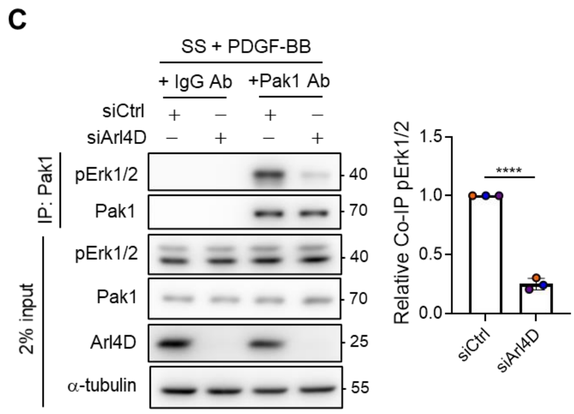

Reply to Reviewer #2

Reviewer #2 (Remarks to the Author):

The authors provide convincing and very interesting data to support the notion that Arl4D works as a scaffolding protein. This function enables activated Erk1/2 to target Pak1 for phosphorylation, an important determinant of cell migration. One of the most interesting features of the study is that scaffolding proteins are required for translocation of Erk from the plasma membrane to other

© 2025. Published by The Company of Biologists under the terms of the Creative Commons Attribution License (https://creativecommons.org/licenses/by/4.0/).

sites in the cell. The advance in this paper is that Arl4D acts as a scaffold protein in order that activated Erk1/2 can target Pak1 at the plasma membrane. The more general idea that is interesting in this paper is that scaffolding is a requirement for substrate targeting by Erk.

The paper is a very good example of concise and impactful writing. The biochemical assays are clear and cleanly conducted and the lay-out of the figures makes it easy to see what is going on with the proposed scaffolding function. The one caveat that this reviewer found with the paper is that the microscopy which is intended to show enrichment of certain proteins at the plasma membrane, and which is important for the demonstration of the idea of membrane targeting and enrichment, does not actually seem to show the expected enrichment and the data visual data do not seem to align well with the Pearson correlation coefficient. Notably, some of the plasma membrane imaging is clear (e.g., Supplementary figures S2A, S4B) while in the main figures, the enrichment is very difficult to see (Fig. 3A, 4C). If the authors were able to provide more convincing figures, this paper would be improved.

Response:

(1) We appreciate the reviewer's comments and have replaced part of Figures 3A and 4C as requested. We have replaced the 10-minute image in Figure 3A to show a stronger correlation with our quantitative data. We did not change the 5-minute images because they are meant to reflect an intermediate stage of recruitment to the plasma membrane.

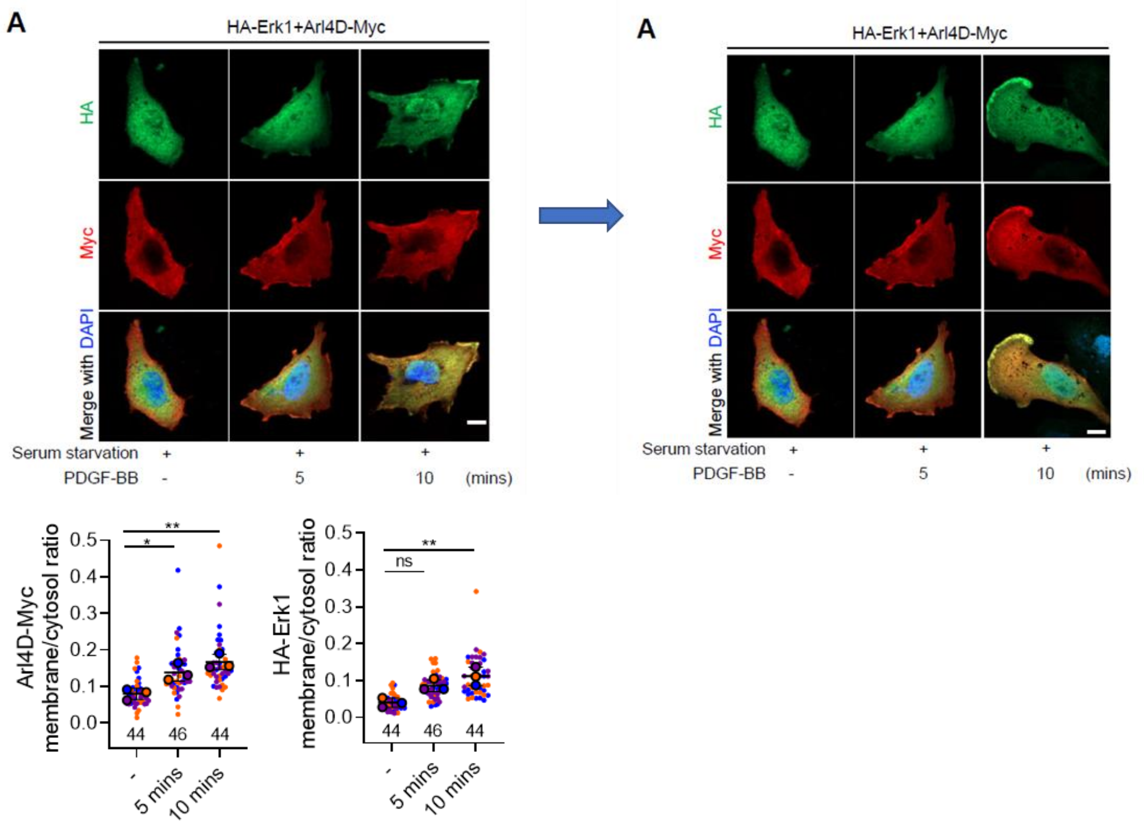

(2) We have replaced a panel in original Figure 4C (now Figure 4D in the revised manuscript) to emphasize the loss of Erk1 plasma membrane signal in the absence of Arl4D. Our previous study showed that Pak1 plasma membrane localization is regulated by the Arl4 family, which creates a positive feedback loop that promotes mutual recruitment to the plasma membrane (Chen, Chiang et al., 2020). Therefore, knocking down Arl4D also partially reduces Pak1's localization at the membrane. For Figure 4C (now Figure 4D), we would like to clarify why the plasma membrane localization sometimes could not ideally align with the Pearson's correlation coefficient. Pearson's correlation tends to judge whether the distribution of a signal forms a positive or negative correlation with another distribution of a signal. Under serum starvation, both Erk1 and Pak1 are diffusely distributed within the cytosol, which makes their colocalization status ambiguous. In contrast, the Pearson's correlation coefficient, when knocking down of Arl4D, successfully

© 2025. Published by The Company of Biologists under the terms of the Creative Commons Attribution License (https://creativecommons.org/licenses/by/4.0/).

distinguished the loss of Erk-Pak1 colocalization at the plasma membrane. This result supports that Erk1 requires Arl4D to target Pak1 at the plasma membrane.

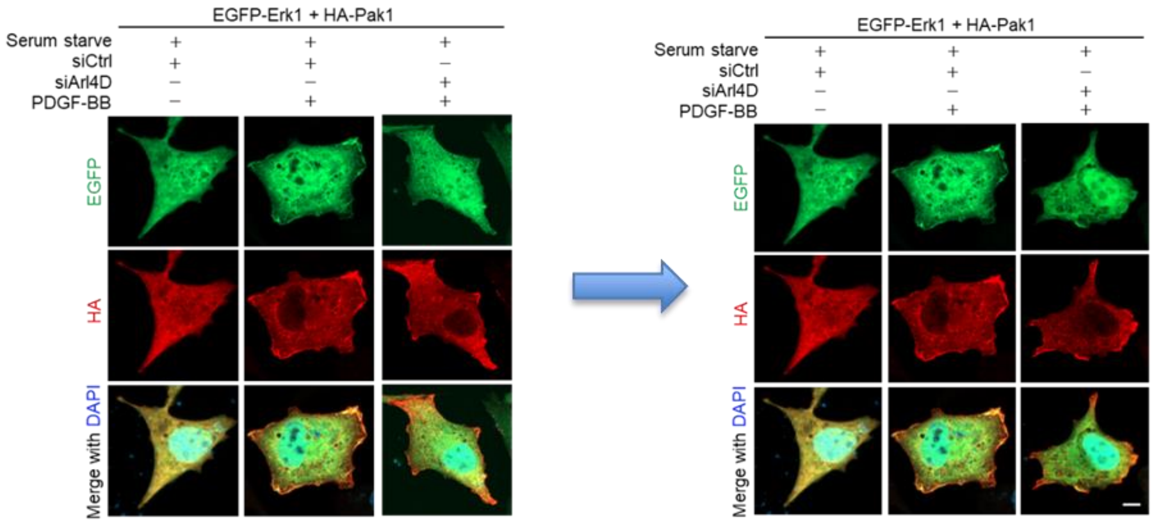

Second decision letter

MS ID#: jcs.263812R1

MS Title: The phosphorylation of Pak1 by Erk1/2 for cell migration requires Arl4D acting as a scaffolding protein

Authors: Ting-Wei Chang; Ming-Chieh Lin; Chia-Jung Yu; FANG-JEN S LEE

Article Type: Research Article

Dear Dr Lee,

I am happy to tell you that your manuscript has been accepted for publication in Journal of Cell Science, pending standard publication integrity checks.

Reviewer 1

Advance summary and potential significance to field

The authors have improved the manuscript and it does advance the field

Comments for the author

None. I think that the paper should be published as they have now presented it.

© 2025. Published by The Company of Biologists under the terms of the Creative Commons Attribution License (https://creativecommons.org/licenses/by/4.0/).
